# Cerebral sterile inflammation in neurodegenerative diseases

**DOI:** 10.1186/s41232-020-00137-4

**Published:** 2020-12-08

**Authors:** Kento Otani, Takashi Shichita

**Affiliations:** 1 Stroke Renaissance Project, Tokyo Metropolitan Institute of Medical Science, 2-1-6 Kamikitazawa, Setagaya-ku, Tokyo, 156-8506 Japan; 2 Division of Biochemistry, Faculty of Pharmacy and Graduate School of Pharmaceutical Science, Keio University, Tokyo, 105-8512 Japan; 3 Precursory Research for Innovative Medical Care (PRIME), Japan Agency for Medical Research and Development (AMED), Tokyo, 100-0004 Japan

**Keywords:** Neuroinflammation, Alzheimer’s disease, Parkinson’s disease, Amyotrophic lateral sclerosis

## Abstract

Therapeutic strategies for regulating neuroinflammation are expected in the development of novel therapeutic agents to prevent the progression of central nervous system (CNS) pathologies. An understanding of the detailed molecular and cellular mechanisms of neuroinflammation in each CNS disease is necessary for the development of therapeutics. Since the brain is a sterile organ, neuroinflammation in Alzheimer’s disease (AD), Parkinson’s disease (PD), and amyotrophic lateral sclerosis (ALS) is triggered by cerebral cellular damage or the abnormal accumulation of inflammatogenic molecules in CNS tissue through the activation of innate and acquired immunity. Inflammation and CNS pathologies worsen each other through various cellular and molecular mechanisms, such as oxidative stress or the accumulation of inflammatogenic molecules induced in the damaged CNS tissue. In this review, we summarize the recent evidence regarding sterile immune responses in neurodegenerative diseases.

## Background

Inflammation is an important biological process in the pathologies of CNS. The cell death induced by various brain pathologies such as neurodegenerative diseases, ischemia, hemorrhage, trauma, and so on triggers inflammation. Conversely, inflammation in the peripheral organs or central nervous system can induce brain cell death. Some reports have demonstrated the relationships between the intestinal microbiome and cerebral inflammation. For example, gut microbiome dysbiosis is related to the dysregulation of the gut-brain axis and supports the neuroinflammatory responses in the brain, leading to the enhanced pathophysiology of AD [1]. Inflammation and brain pathologies thus worsen each other through various cellular and molecular mechanisms, leading to a poor prognosis in terms of quality of life.

The brain consists of various cells, including neurons, astrocytes, microglia, oligodendrocytes, and endothelial cells. If cerebral pathologies exist, all of these kinds of cells are implicated in cerebral inflammation. Neurons, astrocytes, and oligodendrocytes are activated by pathological insults and can become inflammatogenic factors. For example, oxidative stress within brain cells causes neurodegeneration in AD, PD, and ALS [2]. Brain trauma or ischemia induces oxidative stress in brain cells [3]. These oxidative stresses induce the production of reactive oxygen species (ROS) from damaged brain cells, which enhance the vascular dysfunction and the infiltration of circulating immune cells [4]. ROS loosen the cerebral vasculature by decreasing the tight junction proteins and activating matrix metalloproteinases (MMPs) in endothelial cells, resulting in the disruption of the blood-brain barrier (BBB) which causes the infiltration of circulating immune cells and inflammatory factors into the brain [5]. Oxidative stress also induces the production of inflammatory cytokines in macrophages [6].

The cell death of brain cells triggers inflammation. Brain trauma and ischemia directly injures brain tissue and induces the necrotic cell death of various brain cells. Neurodegeneration-related inflammation and cellular stresses also cause neuronal and glial cell death. This releases intracellular components into the extracellular space that triggers inflammation by activating immune cells [7, 8]. Uric acid and adenosine triphosphate (ATP) released from injured mammalian cells trigger the maturation of dendritic cells and subsequent immune responses of CD4- or CD8-positive T cells [9, 10]. ATP is well known as a “find me” signal that is released from injured neurons and astrocytes. Extracellular ATP induces chemotaxis and the production of inflammatory cytokines and ROS in microglia through the activation of GTP-binding protein-coupled P2 receptors [11, 12]. Among various P2 receptors, P2Y12 receptors were recently shown to play pivotal roles in neuroprotection and the maintenance of neuronal activities by adjacent microglia [13]. Extracellular ATP release from astrocytes also regulates the susceptibility to chronic social defeat and modulates depressive-like behaviors, indicating the astrocytic ATP release-dependent regulation of neuronal function against social stresses [14]. Thus, ATP functions as a key molecule for not only the maintenance of neural function but also as the trigger of inflammation in pathologic brain conditions, which may be regulated by extracellular levels of ATP released from brain cells or the expression levels of P2 receptors in microglia. ATP also modifies the function of endothelial cells and myeloid cells other than microglia. The extracellular release of ATP induces the expression of MMP9 and IL-1β in injured brain tissue [15]. These inflammatory molecules promote the disruption of BBB through the degradation of tight junction proteins and endothelial cell death [16]. BBB disruption promotes the infiltration of circulating immune cells in the injured brain. ATP also triggers inflammation by these infiltrating immune cells. Importantly, ATP is an activator of the inflammasome complex that induces the production of IL-1β and IL-18 via caspase-1 activation and pyroptotic cell death [17]. IL-1β contributes to neuronal cell death by increasing the entry of calcium ion through N-methyl-D-aspartate (NMDA) receptor [18]. Inflammasome activation has been reported to be implicated in the pathologies of various CNS diseases, including AD, PD, ALS, and stroke [19–22]. Molecules associated with neurodegeneration and demyelination, such as lysophosphatidylcholine, also activate the inflammasome [23]. Although inflammasome activation occurs in both infiltrating immune cells and neurons and other glial cells [24], the establishment of the inflammatory environment generated by inflammasome activation and the subsequent pyroptotic cell death exaggerates the neuronal dysfunction and neurodegenerative CNS pathologies [25].

In addition to the extracellular release of ATP, some endogenous-specific molecules released from damaged brain cells trigger inflammation. These molecules, called damage-associated molecular patterns (DAMPs), transmit the damage signal to brain cells around the lesion and induce responses for the adaptation to pathological conditions. DAMPs activate immunological receptors in cerebral myeloid cells and other brain cells to produce inflammatory factors. Microglia are the resident cerebral myeloid cells derived from the yolk sac that infiltrate the brain around embryonic day 7 [26]. Microglia and infiltrating macrophages and neutrophils are major producers of inflammatory cytokines. Pattern recognition receptors (PRRs) are important immunological receptors for the recognition of DAMPs released from damaged brain cells. Toll-like receptors (TLRs), receptors for advanced glycation end products (RAGE), and macrophage-inducible C-type lectin (Mincle) are implicated in the triggering or promotion of cerebral inflammation. For example, the accumulation of amyloid β or α-synuclein is thought to cause neurodegeneration in Alzheimer’s disease and Parkinson’s disease, respectively. Amyloid β peptide (Aβ) and α-synuclein are recognized by TLRs to induce neuroinflammation, which promotes glial scar formation and neurodegeneration [27, 28]. The activation of TLR4 signaling in the neuron which is likely responsible for the accumulation of Aβ increases the further expression of Aβ in the neuron and results in the progression of AD [29]. Traumatic or ischemic brain injury induces the extracellular release of DAMPs from damaged brain cells. Mitochondrial DNA and formylated peptides, high mobility group box 1 (HMGB1), and peroxiredoxin family proteins (PRXs) are DAMPs that trigger neuroinflammation through the activation of TLRs and RAGE in cerebral myeloid cells [30, 31]. These inflammatogenic pathways trigger the production of inflammatory cytokines, such as IL-12 and IL-23, which induce the subsequent chronic inflammation mediated by lymphocytes.

**Fig. 1:**
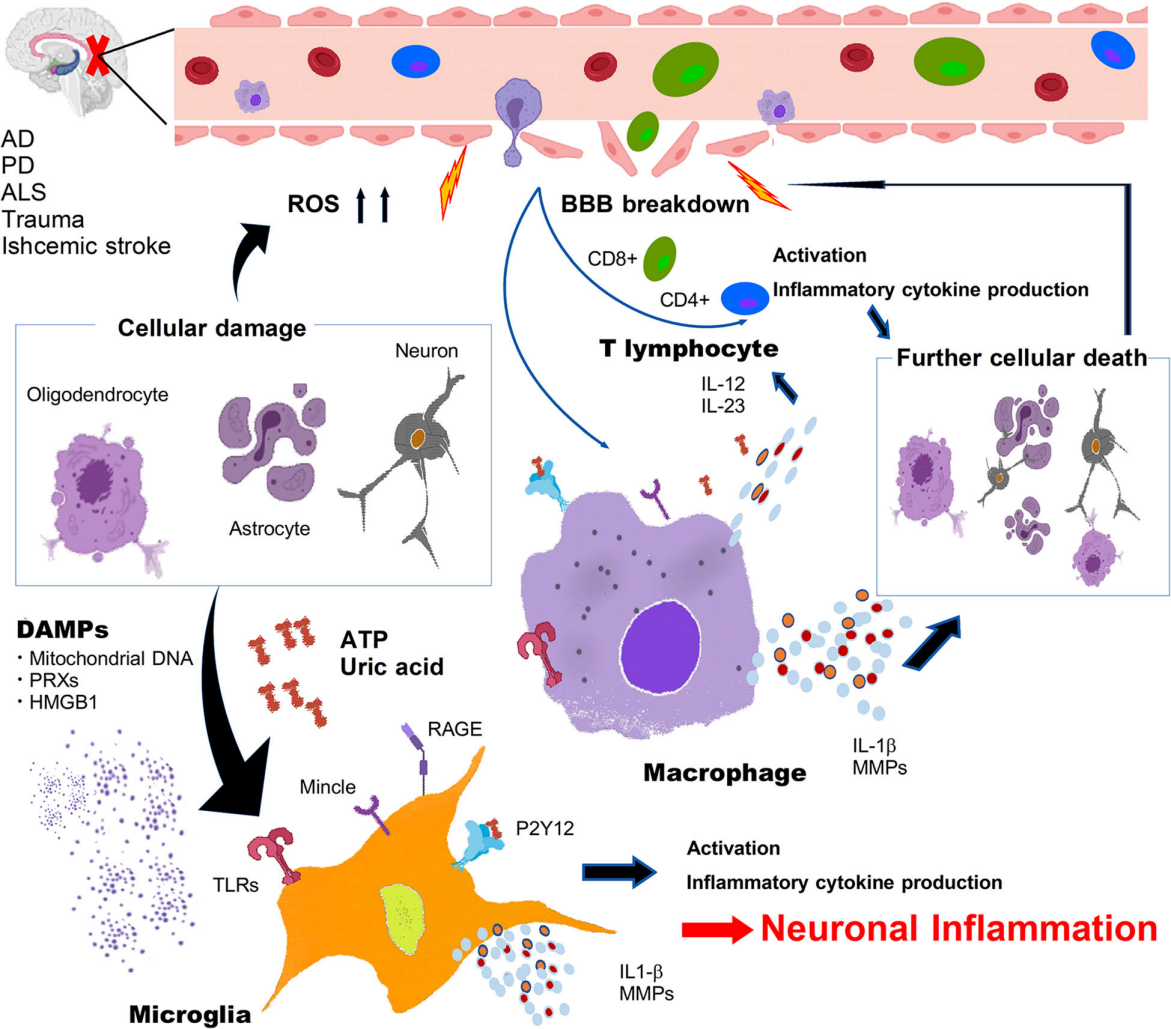
Induction of sterile neuroinflammation. ATP, uric acid, and other DAMPs released from the damaged cells activate microglia and macrophages infiltrating through disrupted BBB. Activated microglia and macrophages produce inflammatory cytokines including the activator of infiltrating T lymphocytes, resulting in the sustained neuroinflammation that leads to further cell death and progression of CNS pathologies

Cerebral inflammation worsens CNS pathologies and causes further generation of inflammatogenic molecules and extracellular DAMPs from damaged brain cells. It is possible that chronic neurodegenerative diseases are caused by this vicious cycle between inflammation and brain damage. Recent studies have clarified the importance of immune responses and immunological receptors in the clearance of inflammatogenic molecules and extracellular DAMPs from the damaged brain. For example, the immune checkpoint blockade against programmed death-1 (PD-1) promotes the clearance of Aβ through the enhanced interferon (IFN)-γ-mediated systemic immune responses in the murine model of Alzheimer’s disease [32]. Currently, therapeutic strategies for regulating neuroinflammation are expected in the development of novel therapeutic agents to prevent CNS pathologies. An understanding of the detailed molecular and cellular mechanisms of neuroinflammation in each CNS disease is necessary for the development of therapeutics (Fig. 1).

## Alzheimer’s disease

AD is the most frequent cause of dementia, which is characterized by progressive cognitive impairment. The temporal and parietal lobes, the hippocampal region in particular, become markedly atrophic in patients with ADs. This is due to the loss of neurons and synapses as a result of progressive neurodegeneration. The accumulation of Aβ and the deposition of neurofibrillary tangles composed of aggregated tau protein have been considered the major pathogenic cause of AD (the amyloid cascade hypothesis) although the cause of these histopathologies has not yet been clarified. Inflammation is also implicated in the pathogenic process of AD, given that the extracellular deposits of Aβ trigger neuroinflammation, and this inflammatory response can promote the intracellular aggregation of tau within vulnerable neurons [33]. Microglia are the primary cells that drive neuroinflammation and gliosis around Aβ plaques with reactive astrocytes [34, 35]. Some of the immunological receptors that interact with Aβ are expected to be important to the inflammatory responses and disease progression in AD pathologies. Indeed, recent studies have identified several receptors in microglia that are strongly related to neuroinflammation in AD. TLR4, RAGE, CD36, and triggering receptor expressed on myeloid cells 2 (TREM2) are receptors for Aβ and induce the production of inflammatory cytokines, complement components, and chemokines [36–38]. Microglia also function as phagocytic cells that remove pathogenic molecules and aggregated proteins, indicating that microglia-related inflammation and the removal of inflammatogenic molecules may be the keys to understanding the AD pathologies.

The normal function of BBB is important for the cerebral accumulation of Aβ, since BBB controls Aβ entry from plasma and Aβ clearance from the brain [39]. Aβ is recognized by RAGE expressed in endothelial cells, and this RAGE-Aβ interaction disrupts BBB by promoting the degradation of tight junction proteins and the secretion of MMPs [40]. Although BBB disruption is implicated in the pathogenesis of AD, Aβ-mediated RAGE activation in microglia also contributes to the vulnerability of endothelial cells and synaptic dysfunction [41, 42]. Thus, RAGE is a multiligand receptor, which was initially identified as a receptor for advanced glycation end products (AGEs) for the induction of neuroinflammation in AD, leading to accelerated neuronal damage and BBB dysfunction [43].

CD36, which is a class B scavenger receptor that is expressed in microglia and endothelial cells, is also a receptor for fibrillar Aβ [44, 45]. Inflammatory responses in microglia stimulated by fibrillar Aβ are largely dependent on CD36, given that CD36-deficient microglia produce much fewer inflammatory cytokines, chemokines, and ROS than microglia from wild-type mice do when microglia are treated with fibrillar Aβ [46]. Aβ-mediated CD36 activation was also reported to generate the senescence-associated secretory phenotype (SASP) [47]. These studies revealed that CD36 bound to Aβ induces the recruitment of microglia and the production of inflammatory mediators in the AD brain.

Owing to recent genome-wide association studies (GWASs), TREM2 has been identified as a receptor associated with a high risk of developing AD; a substitution mutation, the R47H variant of TREM2, is highly associated with AD [48, 49]. TREM2, which is expressed by microglia and neurons in the brain, is important for triggering phagocytosis and inflammatory responses [50, 51]. R47H mutation of TREM2 impairs microglial activation and the microgliosis around Aβ plaque [52, 53]. TREM2 binds directly to Aβ oligomer and plays a pivotal role in the degradation of Aβ through the proteasome pathway within microglia [54, 55]. Single-cell transcriptional profiling of microglia associated with AD revealed the important role of TREM2 in the upregulation of genes related to phagocytosis and lipid metabolism [56]. The possibility of TREM2 actions downstream of CD33, another microglial receptor associated with a high risk of AD, has been demonstrated, which regulates IL-1β-mediated inflammatory cascades in AD pathologies [57].

## Parkinson’s disease

PD is a slowly progressive neurodegenerative disease that mainly impairs the motor system controlling muscle tonus. This is due to the loss of dopaminergic neurons in the substantia nigra. Lewy bodies observed in brain lesion sites are the defining characteristics of PD and are deposits made of a filament structure containing α-synuclein [58]. The abnormal accumulation of α-synuclein in the brain, which is caused by *Snca* gene polymorphisms, dysfunction of a protein degradation system such as proteases, autophagy, or the ubiquitin-proteasome system, is considered to be the major cause of PD pathologies [59–61]. The aggregation of α-synuclein is dependent on a seeding-nucleation mechanism that leads to fibril growth [62]. Although the intracellular aggregation of α-synuclein protein is the pathological characteristic of PD, α-synuclein pathologies are considered to propagate from neuron to neuron, as in prion disease [63].

Extracellular α-synuclein is an inflammatogenic molecule that contributes to the progression of neuroinflammation and pathologies in PD [64, 65]. Fibrillar α-synuclein activates TLR2 and the nucleotide oligomerization domain-like receptor pyrin domain containing 3 (NLRP3) inflammasome to produce IL-1β, which is a major cytokine implicated in the initiation and progression of PD [66–69]. Increased expression levels of IL-1β and NLRP3 are observed in the serum of patients with PD [70]. Nitrated α-synuclein triggers both microglia-mediated neuroinflammation and lymphocyte-mediated acquired immune responses that worsen PD pathologies [71, 72]. Microglial TLR4 is also activated by soluble or fibrillar α-synuclein to induce the production of inflammatory cytokines, chemokines, and ROS [73]. Among chemokines, CXCL12 is important for the accumulation of microglia activated by α-synuclein [74].

PTEN-induced putative kinase 1 (PINK1) and PARKIN have been identified as causal genes associated with familiar PD [75, 76]. PINK1, a serine/threonine kinase, generates phosphorylated ubiquitin, which induces the full activation of the ubiquitin ligase activity of PARKIN [77]. PINK1 and PARKIN are important for the removal of damaged mitochondria through mitophagy and the prevention of neuroinflammation, since mitochondrial damage causes the extracellular release of mitochondrial DAMPs [78]. Mitochondrial DNA stress induces the activation of the cGAS-STING pathway [79]; in fact, a deficiency of STING recovers the loss of dopaminergic neurons in the substantia nigra of aged Parkin-deficient mice. Although the relationships between mitochondrial dysfunction and α-synuclein accumulation remain to be clarified, two recent reports suggest the important roles of α-synuclein in the regulation of mitochondrial function [80, 81]. Thus, α-synuclein-mediated neuroinflammation is essential for the generation of PD pathologies.

## Amyotrophic lateral sclerosis

ALS is a slowly progressive neurodegenerative disease. Neurodegeneration in ALS impairs motor neuron function in the CNS [82]. The accumulation of rounded or thread-like deposits containing TAR DNA binding protein 43 (TDP-43) is considered to play a pivotal role in the progression of ALS pathologies [83]. The inclusion body generated by the accumulation of abnormally phosphorylated TDP-43 is important to the progression of ALS [84, 85]. Recently, shortened TDP43 splice variants have been demonstrated to preferentially accumulate within neurons and exert a neurotoxic effect, leading to progressive neurodegeneration in ALS [86]. The extracellular TDP-43 activates microglia through the CD14 receptor and enhances proinflammatory cascades such as NF-κB and AP-1 pathways, which are involved in the neurotoxicity of motor neurons [87].

Many studies have demonstrated that neuroinflammation induced by activated microglia and T lymphocytes is implicated in the progressive pathologies of ALS [88]. Microglia and astrocytes play an important role in ALS pathogenesis, given that cellular damage induced by mutant superoxide dismutase (SOD1) in microglia and astrocytes exaggerates the ALS pathologies [89, 90]. The accumulation of misfolded SOD1 induces inflammasome activation and IL-1β production, which is implicated in the progression of ALS pathologies [91]. The progressive and spreading motor neuron pathologies in ALS seem to be due to the propagation of misfolded proteins such as SOD1 [92]. The intracellular aggregation of misfolded SOD1 induces the production of inflammatory cytokines in microglia [93]. The administration of antibodies against misfolded SOD1 attenuates the ALS pathologies, indicating that the aggregation of misfolded SOD1 is one of the pivotal mechanisms causing motor neuron degeneration [94].

The infiltration of helper (CD4+) and cytotoxic (CD8+) T lymphocytes is observed in the neurodegenerative lesions of ALS [95]. IFN-γ-producing helper T (Th1) and IL-17-producing helper T (Th17) lymphocytes are inflammatory subtypes detected in the blood and cerebrospinal fluid of ALS [96, 97]. The deficiency in T lymphocyte function in the ALS model enhances the protection of motor neurons and attenuates the glial activation, however, suggesting the neuroprotective role of T lymphocytes in ALS pathologies [88, 98]. IL-4-expressing regulatory T cells attenuate the activation of inflammatory microglial cells and prevent the progression of ALS pathologies [99]. Thus, the accumulation of inflammatogenic factors induces the activation of glial cells. T lymphocyte-mediated regulation of glial inflammatory responses is pivotal for the progression of ALS pathologies.

## Conclusion

**Fig. 2:**
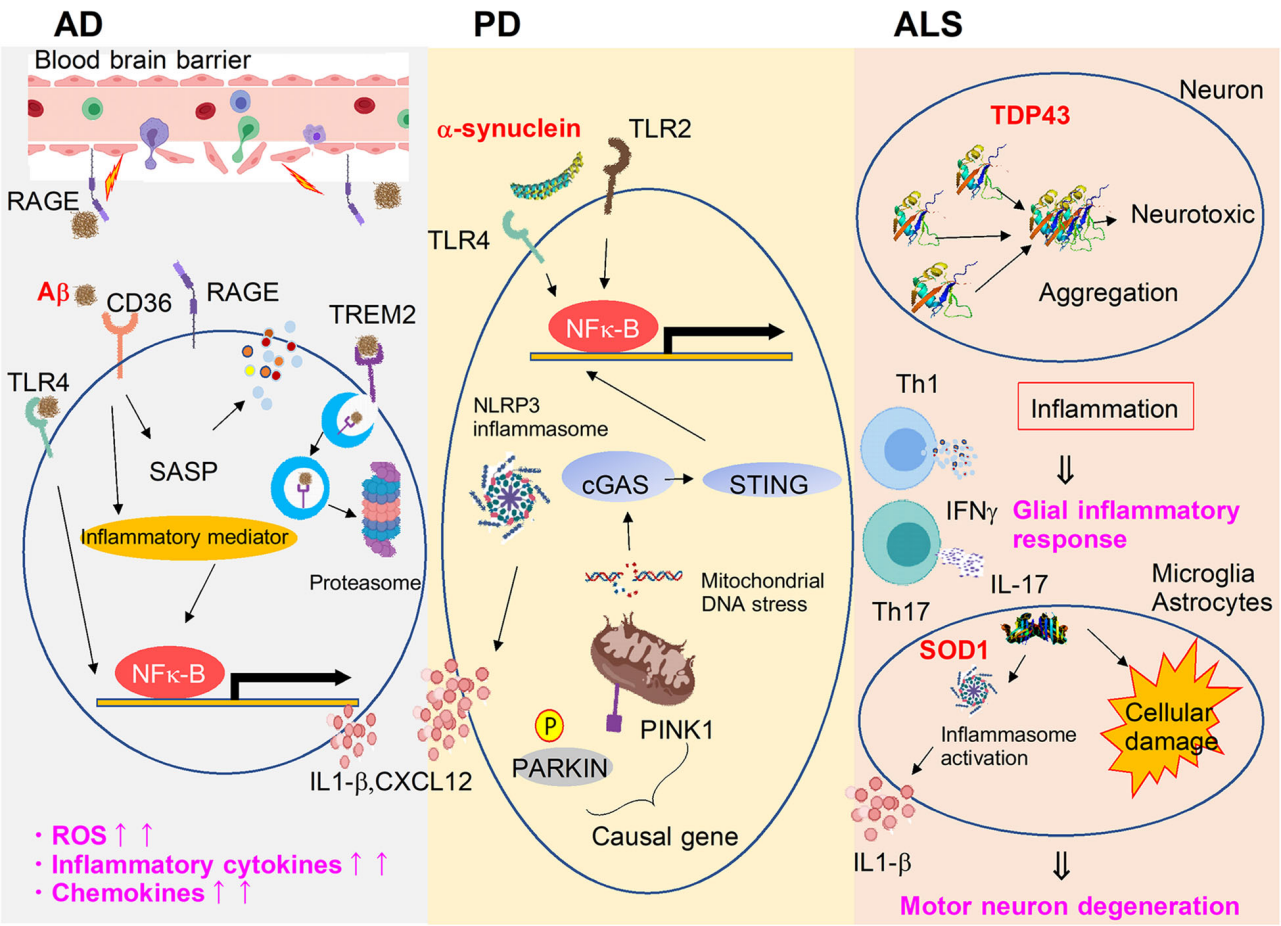
Different mechanisms of neuroinflammation among AD, PD, and ALS. This is the summarized figure of molecular and cellular mechanisms of sterile neuroinflammation in AD, PD, and ALS. Different cells and molecules regulate the neuroinflammation that has a pivotal role in the induction of each CNS pathology

Neuroinflammation plays an important role in the progression of neurodegenerative diseases. The generation of inflammatogenic molecules due to the cellular damage or pathologies of neurodegenerative diseases induces inflammation by glial cells and immune cells. The clearance of these inflammatogenic molecules from brain lesions is also a result of the function of the glial or immune cells implicated in neuroinflammation. Thus, the immune-neural network in the pathologies of neurodegenerative diseases is complicated (Fig. 2). Finding the pathway that removes proinflammatory factors or promotes neural repair without causing inflammation will be necessary for developing therapeutic methods to regulate the inflammation in the pathophysiology of neurodegenerative diseases. Identification of the key molecules that regulate the inflammation and neural repair to improve CNS pathologies is important to the development of therapeutic methods for CNS diseases.

## Data Availability

Not applicable.

## Abbreviations

CNS: Central nervous system
AD: Alzheimer’s disease
PD: Parkinson’s disease
ALS: Amyotrophic lateral sclerosis
ROS: Reactive oxygen species
MMPs: Matrix metalloproteinases
BBB: Blood-brain barrier
ATP: Adenosine triphosphate
DAMPs: Damage-associated molecular patterns
PRRs: Pattern recognition receptors
TLRs: Toll-like receptors
RAGE: Receptors for advanced glycation end products
Mincle: Macrophage-inducible C-type lectin
HMGB1: High mobility group box 1
PRXs: Peroxiredoxin family proteins
Aβ: Amyloid beta
TREM2: Triggering receptor expressed on myeloid cells 2
AGEs: Advanced glycation end products
SASP: Senescence-associated secretory phenotype
GWASs: Genome-wide association studies
NLRP3: Nucleotide oligomerization domain-like receptor pyrin domain containing 3
PINK1: PTEN-induced putative kinase 1
TDP43: TAR DNA binding protein 43
SOD1: Superoxide dismutase
